# Validity and Practical Application of Muscle Oxygenation Monitoring for Identifying Maximal Fat Oxidation in Cyclists

**DOI:** 10.1002/ejsc.70025

**Published:** 2025-07-22

**Authors:** Ander Romarate, Aitor Pinedo‐Jauregi, Andri Feldmann, Aitor Viribay, Jordan Santos‐Concejero

**Affiliations:** ^1^ Emen4sport Leioa Spain; ^2^ Department of Physical Education and Sport University of the Basque Country UPV/EHU Vitoria‐Gasteiz Spain; ^3^ Society, Sports, and Exercise Research Group (GIKAFIT) Department of Physical Education and Sport Faculty of Education and Sport University of the Basque Country UPV/EHU Vitoria‐Gasteiz Spain; ^4^ Research Group in Physical Activity, Physical Exercise and Sport (AKTIBOki) Department of Physical Education and Sport Faculty of Education and Sport University of the Basque Country UPV/EHU Vitoria‐Gasteiz Spain; ^5^ Institute of Sport Science University of Bern Bern Switzerland; ^6^ Glut4Science Physiology Nutrition and Sport Vitoria‐Gasteiz Spain; ^7^ Institute of Biomedicine (IBIOMED) University of León León Spain; ^8^ Virtual Sensorization Research Group ‐ Visens (IT‐1381‐2019), Department of Physical Education and Sport, Faculty of Education and Sport University of the Basque Country UPV/EHU Vitoria‐Gasteiz Spain

**Keywords:** incremental exercise test, metabolic analysis, near‐infrared spectroscopy, physiology, wearable

## Abstract

The accurate detection of several physiological milestones, such as maximal fat oxidation (MFO), is an important factor for cycling performance and for programming effective and individualised training. However, the procedure to identify the MFO is often too complex and expensive. Near‐infrared spectroscopy (NIRS) technology provides a noninvasive measurement that can be used to detect different physiological variables. The aim of this study was to assess the validity of utilising the muscular oxygen saturation visualisation methodology for the identification of the MFO point in trained cyclists. Twenty‐two recreational endurance‐trained cyclists (19 men and 3 women; age: 27.9 ± 5.4 years; body mass: 69.7 ± 7.1 kg and VO_2max_: 60.3 ± 7.0 mL/kg/min) performed a submaximal and maximal exhaustion test. All the data were collected on a single day. The validity of the visualisation methodology for the maximal fat oxidation point was analysed against a gas analyser. The detection of maximal fat oxidation (MFO) using the methodology and device employed does not appear to accurately specify the precise point at which MFO occurs (bias = 90 ± 218 s and LOA = 429 s). However, our results indicate that it may be a valid technique for identifying the MFO zone; biases were HR = 4.7 ± 11.9 bpm, VO_2_ = 1.49 ± 5.7 mL/kg/min and power = 19.5 ± 31.2 W, whereas the concordance coefficients were 0.783, 0.243 and 0.170, respectively. It is not possible to detect MFO using NIRS device. However, it is possible to detect a general zone in which MFO occurs.

## Highlights

- The detection of the maximal fat oxidation point using muscle oxygen saturation plotted as a function of time does not appear to accurately specify the precise point at which maximal fat oxidation occurs.
- It is possible to detect a general zone in which the maximal fat oxidation occurs.
- Near‐infrared spectroscopy technology is useful for coaches without time to do complicated analyses.

## 1 Introduction

Training zones or intensity domains are the most used parameters to design exercise training aimed at performance and health. To determine the intensities associated with each zone, different techniques are used. In most cases, they are determined from physiological changes informed by lactate concentration measurement, dynamics in respiratory outcomes, heart rate behaviour and most recently, by assessing muscle oxygen saturation (Cottin et al. 2007; Pallarés et al. 2016; Keir et al. 2021; Sendra‐Pérez et al. 2023). The accurate detection of these changes is an important factor in understanding cyclist's performance and programming effective and individualised training. For example, lactate threshold, in conjunction with maximal oxygen uptake (VO_2max_) and efficiency, are considered the main determinants of cycling performance (Joyner and Coyle 2008). However, the identification of these physiological variables typically requires costly equipment, including devices such as gas analysers. These procedures are beyond the scope of reasonable application for most coaches and cyclists. In this regard, evaluation methodologies applicable on a daily basis, such as heart rate, heart rate variability (Rogers et al. 2021; Mateo‐March et al. 2022) or near‐infrared spectroscopy (NIRS) (van der Zwaard et al. 2016; Cayot et al. 2021), have been proposed for the detection of biomarkers such as lactate threshold (Farzam et al. 2018) and VO_2max_ (Okushima et al. 2016; Feldmann et al. 2022). NIRS technology provides a noninvasive measurement of the balance between oxygen delivery and consumption in active tissue (Ferrari et al. 2011) and thereby, provides an interesting and practical technique to detect critical exercise thresholds directly in the exercising muscle (van der Zwaard et al. 2016). Despite the existing studies, there is still a need for further research to establish associations between these variables and NIRS technology.

Metabolic outcomes, such as substrate utilisation, explain the bioenergetics of the working muscle and are heavily correlated with the abovementioned physiological determinants. In this regard, it is well know that carbohydrate (CHO) and fat (FAT) oxidation impact on gross efficiency and determines fatigue and durability (Hargreaves and Spriet 2020; MacDougall et al. 2022). The assessment of fat oxidation rates (FATox) serves as an indirect measure of an individual's mitochondrial efficiency and oxidative capacity under exercise stress, which varies across different demographic groups (San‐Millán and Brooks 2018). In this sense, an endurance‐trained population will show greater FATox capacity and lactate clearance at higher absolute exercise intensity, meaning a higher capacity to produce energy through oxidation, when compared to sedentary people or patients with metabolic syndrome (San‐Millán and Brooks 2018). Therefore, measuring and monitoring FATox as a marker of total oxidative capacity hints the exercising capacity of a subject. Such measurements are integral for developing tailored training and nutritional strategies aimed at optimising FATox to enhance exercise performance and health outcomes. In fact, the rate of maximal fat oxidation (MFO) and the specific intensity range at which it occurs are crucial for effective weight management, with implications for cardiovascular and metabolic health (Wang et al. 2015; Dandanell, Boslev Praest et al. 2017; Dandanell, Husted et al. 2017; Chávez‐Guevara et al. 2020), and they also correlate with improved endurance performance (Frandsen et al. 2017). Traditionally, MFO is quantified using indirect calorimetry, a gold‐standard method for evaluating metabolic efficiency (Purdom et al. 2018; Amaro‐Gahete et al. 2019). However, the high day‐to‐day variability in MFO necessitates repeated measures for accurate determination, which poses challenges for its practical application as a metabolic biomarker in clinical and athletic settings (Chrzanowski‐Smith et al. 2020). Recent advances in metabolic testing may provide more accessible and reliable means of assessing MFO, suggesting a need for ongoing research to refine these methodologies for broader use.

Training intensity has been identified as one of the most important factors in determining substrate oxidation, that is, MFO (Purdom et al. 2018). NIRS has been shown to consistently reflect change in exercise intensity and shifts in exercise intensity domains (Kirby et al. 2021). Specifically, it has been shown that tissue deoxygenation is a function of exercise work rate (Chuang et al. 2002) similar to the findings of MFO (Thompson et al. 2006). In addition, NIRS derived exercise thresholds agree with standard exercise thresholds indicate shifting metabolism such as the lactate threshold (Grassi et al. 1999) and ventilatory threshold or respiratory compensation point (Yogev et al. 2022). It has been observed that exercise intensity generates a clear pattern of substrate utilisation as it increases (Purdom et al. 2018). Exercise thresholds help identify the crossover point where substrate utilisation transitions between fat oxidation and carbohydrate oxidation (Borel et al. 2015; Purdom et al. 2018). The general mechanism by which NIRS could be useful in determining substrate utilisation, such as the crossover point or MFO, lies in the fact that oxygenation measured by NIRS, in terms of the balance between oxygen supply and demand, is tightly coupled to metabolic rate (Bellotti et al. 2013; Azevedo et al. 2020). The capability to sustain a net zero change (i.e., a rate of change equal to zero) implies insights into metabolic rate concerning substrate oxidation versus oxygen supply. As ATP and metabolic rate increase, this rate becomes negative as oxygen demand exceeds supply (Boyette and Manna 2022). Although fat oxidation has a higher overall ATP yield, the limited capacity per unit of time to generate ATP drives the need for carbohydrate utilisation (Spriet 2014; Purdom et al. 2018). Therefore, as the metabolic rate increases, fat oxidation is substituted in favour of carbohydrate oxidation (Maunder et al. 2018). Detecting this bioenergetic shift according to external intensity (pace, power, etc.) in athletes or patients represents an opportunity to improve their performance and metabolic health.

Measuring muscle oxygen saturation (SmO_2_) is a noninvasive methodology that can detect changes in the balance between oxygen delivery and oxygen consumption in the muscles during exercise (Vasquez‐Bonilla et al. 2021). As a result, many studies have used NIRS to detect different physiological breakpoints (Rodrigo‐Carranza et al. 2021; Salas‐Montoro et al. 2022). Farzam et al. (2018) try to detect lactate threshold using NIRS technology. They showed that the Humon (Humon Beta, Dynometrics Inc.) device is valid for detecting lactate threshold power (when athlete’s lactate blood concentration reaches 4 mmol/L) with a difference of ± 21.4 W and less than 3 min compared to the invasive method. In another study, the authors compared maximal lactate steady state (MLSS) with detection using NIRS technology and concluded that MLSS can be accurately detected using NIRS technology (Bellotti et al. 2013). Zurbuchen et al. (2020) found that higher MFO values are associated with a slower slope or rate of muscle deoxygenation indicating better matching of O_2_ delivery and consumption, alongside a higher overall muscle deoxygenation and rightward shift of a deoxygenation breakpoint. A greater rate of muscle deoxygenation is associated with a higher VO_2max_ (Okushima et al. 2016), further underpinning a potential application of NIRS to assess MFO.

The primary aim of this study was to assess the validity of utilising the SmO_2_ visualisation methodology for the identification of the MFO point in trained cyclists. It was hypothesised that the MFO point could be determined by observing changes in SmO_2_ over time during a laboratory‐based cycling test.

## 2 Material and Methods

### 2.1 Participants

Twenty two recreational cyclists (19 men and 3 women; age: 27.9 ± 5.4 years; VO_2max_: 60.3 ± 6.99 mL/kg/min; weight: 69.65 ± 7.07 kg and 8 ∑Folds: 266.3 mm) participated in this study. All participants had more than 2 years of experience in endurance sports and performed more than 10 h per week of endurance training. This study was approved by the Institutional Research Ethics Committee (Cod. CEISH‐113/2019), and all participants provided written informed consent, as outlined in the Declaration of Helsinki (Holm 2013).

### 2.2 Procedures

All the data were collected on a single day in the laboratory. The participants followed their usual diet the days prior to the test. 48 h prior to the assessment, the participants did not perform moderate or high intensity training and the 24 h prior the participants only performed low‐intensity training or rest (they refrained from strength training). Four hours before the start of the test, the participants did not ingest any caloric or stimulant food or drinks. Before the test session, all participants performed a standardised warm‐up protocol consisting of 10 min at 50–75 w pedalling at a cadence of 80–90 rpm.

#### 2.2.1 MFO and VO_2max_ Test

Each participant performed the test with their personal bike installed on a stationary Tacx‐Trainer device (Tacx Neo Smart T2800, Wassenaar, Netherlands) and associated software (Tacx Trainer software 4, Wassenaar, Netherlands). The device was calibrated before each test as suggested by the manufacturer. After the warm‐up, participants performed an incremental test starting at 75 w (for women) and 100 w (for men) with increments of 25 w (for women) and 35 w (for men) every 4 min. When the respiratory exchange ratio (RER) value reached 1.0, the participants stopped pedalling and rested for 15 min. During the rest period, the participants consumed a drink containing 35 g CHO (Carbolider, Fullgas Sport, Astigarraga, Spain). Subsequently, for the detection of VO_2max_, participants performed an incremental test to exhaustion starting at the power at which RER = 1.0 was reached, with intensity increments of 25–35w/min to exhaustion. During the test, the subjects were instructed to pedal at a constant cadence between 80 rpm and 90 rpm. The criteria suggested by Howley et al. (1995) were used to confirm VO_2max_. The heart rate was recorded using Polar H10 chest strap (Polar Electro Oy, Kempele, Finland).

### 2.3 Measurements

#### 2.3.1 Anthropometric Measurements

Before the physical test, all participants underwent an anthropometric assessment. Body mass (kg), skinfolds (mm), body perimeters (cm) and body diameters (cm) were measured in each participant. Body mass was obtained using an electronic scale (Seca Instruments Ltd., Hamburg, Germany), and the six skinfolds (triceps, subscapular, suprailiac and abdominal) were measured with a calliper (Harpender Lange, Cambridge, MA, USA) following the indications specified by Society for the Advancement of Kinanthropometry (ISAK). The sum of the six folds was also calculated (∑ fold).

#### 2.3.2 Metabolic Analysis

Gas exchange was measured continuously in the MFO and VO_2max_ test using an Ergocard breath‐by‐breath gas analyser (Ergocard, Medisoft, Sorinnes, Belgium). Breath‐by‐breath recordings of VO_2_ and VCO_2_ were obtained throughout the test, calibrated for room humidity, flow and O_2_/CO_2_ concentration prior to each test. The gas analysers were calibrated using a 4.95% CO_2_–95.05% N_2_ gas mixture. Prior to the calculation of substrate oxidation, a 30 s rolling average was applied to the breath‐by‐breath VO_2_ and VCO_2_ data to reduce variability and improve interpretability of the metabolic responses. Heart rate (HR) with a prostrap (Polar H10, Polar Electro Oy, Kempele, Finland) was recorded throughout the test. At the end of each step, participants were asked for the ratio of perceived exertion using the CR‐10 RPE scale, differentiating respiratory (RPEres) and muscular (RPEmus) exertion (Iturricastillo et al. 2016).

#### 2.3.3 Muscle Oxygen

The NIRS device was placed in the dominant vastus lateralis (VL), and SmO_2_ (Moxy, Fortiori Design LLC, Minneapolis, MN, USA) was measured during the test. The NIRS device was placed at two‐thirds between anterior superior iliac spine and the lateral side of the patella. The sensors were fixed in place using medical adhesive tape and covered with a compatible, commercially available light shield to eliminate possible ambient light intrusion. The sampling rate was set at 2 Hz, which sampled the four wavelengths over 20 cycles for an averaged output every 0.5 s. An emitter‐detector spacing of 25 mm provides a penetration depth of 12.5 m. The tissue thickness of the subjects at the measurement site was 11.6 ± 4.2 mm.

### 2.4 Data Analysis

The NIRS device and gas analyser were synchronised and continuously collected data throughout the duration of the protocols. The NIRS device was connected to a smartwatch (Garmin Forerunner 935), from which SmO_2_ data were extracted from the original ‘fit’ file recorded by the smartwatch. The MFO point was identified using a gas analyser by observing the dynamics of carbohydrate (CHOox) and fat (FATox) oxidation over time. CHOox and FATox data were calculated in grams using the same equation employed by (San‐Millán and Brooks 2018).

$$\text{FATox}\;\left(g\cdot \min^{-1}\right)=1.67{\text{VO}}_{2}\left(L\cdot \min^{-1}\right)-1.67{\text{VCO}}_{2}\left(L\cdot \min^{-1}\right)$$

$$\text{CHOox}\;\left(g\cdot \min^{-1}\right)=4.55{\text{VO}}_{2}\left(L\cdot \min^{-1}\right)–\;3.21{\text{VCO}}_{2}\left(L\cdot \min^{-1}\right)$$

SmO_2_ and substrate oxidation were plotted as a function of time with a 30 s rolling mean for visual detection of the MFO point. The method to detect the breakpoint in SmO_2_ relied on visually identifying the workload that led to a change the linearity in SmO_2_. The exercise intensity at which the highest fat oxidation ratio was observed was established as MFO (Achten et al. 2002). The MFO point values were individually verified by two researchers, with any discrepancies resolved by consensus. The method for detecting the MFO point was blinded.

Also, segmented regression analysis using a fourth filter order was performed. The time SmO_2_ MFO point detected by visual analysis was used as the starting point for the segmented regression. The intensity at the MFO point detected was indicated in terms of power output (PO), VO_2_ and HR. The value of each variable was calculated as the mean of 30 s, 15 s before and after the point at which the MFO was detected using each methodology.

### 2.5 Statistical Analyses

Data were analysed using statistical environment R (RStudio Inc., Boston, MA). Descriptive values were presented as mean and standard deviations (SDs). The validity of the visual SmO_2_ method and segmented regression method was evaluated using Lin’s concordance coefficient (LCC), mean absolute percent error (MAPE) and agreement with the reference method (gas analyser). Agreement plots with 95% limits of agreement (LoA) (mean difference ± 1.96 SD) were generated to visualise the degree of agreement between each method and the reference method (Krouwer 2008). Heteroscedasticity/homoscedasticity was assessed by calculating the Pearson correlation coefficient between absolute differences and reference values. A method was considered valid if the MAPE was less than 10% and the LCC was greater than 0.7. This threshold for MAPE (< 10%) has been commonly used in previous validation studies involving physiological data and wearable devices and represents an acceptable level of agreement between methods (BOUDREAUX et al. 2018; Johnston et al. 2021; Carrier et al. 2025). Significance was set at *p* < 0.05. The correlation was defined as follows: negligible (0.00–0.30), weak (0.30–0.50), moderate (0.50–0.70), strong (0.70–0.90) or very strong (0.90–1.00) (Mukaka 2012).

## 3 Results

**FIGURE 1:**
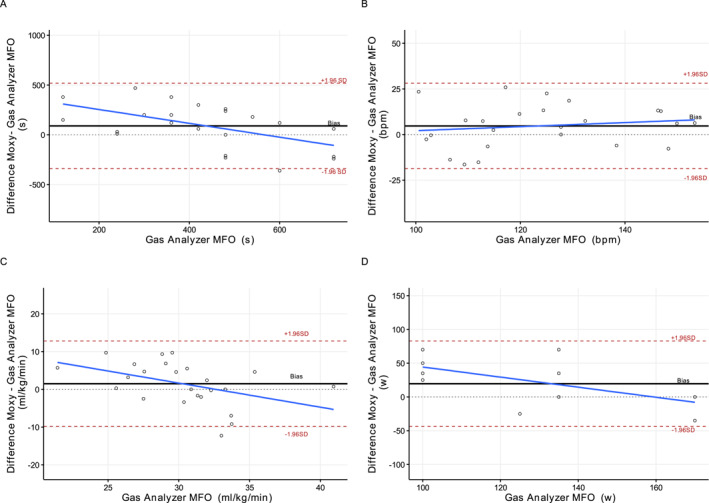
Agreement plots between Moxy device and reference method. Bias: mean bias between the Moxy and the reference method for every participant. Dashed horizontal lines: limits of agreement between the Moxy and the gas analyser MFO method. MFO: maximal fat oxidation.

Figure 1 illustrates the agreement between the reference method and NIRS detection. A degree of bias was observed when comparing time points from each method (bias = 90 ± 218 s and LOA = 429 s). Additionally, low concordance and high MAPE were observed (LCC = 0.23 and MAPE = 58.6%). However, the bias of intensity values was HR = 4.7 ± 11.9 bpm, VO_2_ = 1.49 ± 5.7 mL/kg/min and PO = 19.5 ± 31.2 W, whereas the concordance coefficients were 0.783, 0.243 and 0.170, respectively. The MAPE was 8.6% for HR, 15.9% for VO_2_ and 22.6% for PO. No heteroscedasticity of data were observed (time: *r* = −0.53; HR: *r* = 0.15 and VO_2_: *r* = −0.44), which can be visually evaluated in Figure 1.

Table 1 presents the agreement between the reference method and segmented regression analysis. As can be observed, power, VO_2_ and time were not a valid due to high MAPE from 17.16% to 61%. However, HR was similar between both methods. No heteroscedasticity was observed (time: *r* = −0.79; HR: *r* = 0.17; VO_2_: *r* = −0.36 and PO: *r* = −0.79).

**TABLE 1:** Agreement between the reference method against segmented regression analysis.

Variable	Bias	Bias SD	LOA	LW LOA	UP LOA	LCC	LW LCC	UP LCC	MAPE (%)
Time(s)	42.07	230.17	451.13	−409.06	493.19	−0.09	−0.45	0.30	61.53
HR (bpm)	1.22	11.52	22.57	−21.35	23.79	0.78	0.56	0.90	7.91
VO_2_ (mL/kg/min)	0.52	5.87	11.51	−10.99	12.02	0.29	−0.08	0.60	17.16
Power (w)	7.29	30.07	58.94	−51.65	66.23	0.24	−0.16	0.56	18.70

*Note:* Bias: mean bias between the Moxy and the FatMax method for every participant.

Abbreviations: LCC, Lin’s concordance coefficient; LOA, limits of agreement; LW LCC, lower Lin’s concordance coefficient; LW LOA, low limits of agreement; MAPE, mean absolute percent error; SDs, standard deviations; UP LCC, upper Lin’s concordance coefficient; UP LOA, upper limits of agreement.

## 4 Discussion

The aim of this study was to evaluate the validity of the SmO_2_ visualisation methodology for detecting the MFO point in trained cyclists. Based on our results, the detection of MFO using the methodology and device employed does not appear to accurately specify the precise point at which MFO occurs. However, our results indicate that it may be a valid technique for identifying the range of exercise intensity where MFO happens.

It is well‐established that exercise test protocols play a crucial role in the detection of thresholds (Jamnick et al. 2018; Kouwijzer et al. 2019). In fact, exercise test protocols largely determine exercise thresholds and results, presenting a fundamental challenge when attempting to bridge the gap between training and testing. It has been documented that short stage or ramp protocols lasting no longer than 12 min are recommended for achieving a valid VO_2max_ (Buchfuhrer et al. 1983), whereas longer stage tests are used to determine lactate threshold and steady‐state responses of bioenergetics (Bentley et al. 2007). The duration and intensity of exercise dictate physiological responses, with measurable parameters reacting in a cascading series of events (Burton et al. 2004). Given the proximity of exercise thresholds resulting from different physiological metrics, a complex interaction of bioenergetic pathways is at play. If we consider lactate threshold as a standard in exercise threshold testing, when comparing the first lactate threshold with MFO, data does not fully support their interchangeability, even though both thresholds have often been used synonymously (Ferri Marini et al. 2022). This is despite the clear interaction between lactate and fat oxidation, with lactate acting to either reduce the circulation of free fat acids through the activation of HCAR‐1 or reduce beta oxidation through downregulation of *β*‐ketothiolase (Brooks 2020). The imprecision with which exercise tested thresholds are derived, resulting from highly scripted exercise test protocols when compared to their expected physiological dependency, raises significant questions. Similar questions can be raised when applying new technologies, such as muscle oxygenation, derived from NIRS.

In the study, it is observed that the detection of the specific MFO point using NIRS does not coincide with the MFO point detected through gas analysis (bias 42.07 ± 230.17 s). These results are consistent with studies that have detected the onset of blood lactate accumulation (OBLA) lactate threshold using NIRS (Farzam et al. 2018). In their study, a 2 min and 32 s time difference was obtained between estimations of blood lactate threshold and NIRS technology. However, these authors suggested that when considering power data, there was a bias of 21.4 W in the detection of the OBLA lactate threshold. In line with a previous study, Batterson et al. (2023) observed in a sample of 10 elite soccer players that there were no significant differences between SmO_2_ breakpoints and maximum deviation (D_max_) lactate thresholds 1 and 2. They suggested that the first breakpoint appeared between the first and second D_max_ lactate thresholds. In this regard, our study follows similar results. Although the time data differ, if we compare the measured intensity with HR and VO2 at which the change occurs, we find that NIRS could detect the range of intensity at which MFO occurs (HR bias: 1.22 ± 11.52 bpm and VO_2_ bias: 0.52 ± 5.87 mL/kg/min). Our objective has not been studied in the literature, which makes it difficult to compare with other studies. However, considering other attempts to detect thresholds, as in the previous study (Bellotti et al. 2013), it was also observed that the maximal lactate steady state could be detected using NIRS technology, as they found high correlations in HR and VO_2_ markers detected using NIRS and MLSS. Therefore, the initial hypothesis is not fully fulfilled, as it is not possible to detect MFO point by visualising the dynamics of SmO_2_ over time. Therefore, although the precise localisation of the MFO point was not possible using the applied NIRS methodology, the identification of a general exercise intensity zone where fat oxidation is elevated may still hold some practical value. However, its application should be approached with caution. As shown by Achten and Jeukendrup (2003), even small changes in exercise intensity can cause substantial alterations in fat oxidation rates, complicating the accurate targeting of this metabolic zone. Furthermore, recent work has demonstrated that the intraindividual variability in FatMax determination can be large—even under controlled conditions—thus questioning its reliability for individual exercise prescription (Meyer et al. 2009). Combined with the limited reproducibility of NIRS‐based methods, this further supports the need to interpret the NIRS‐derived MFO zone as an approximate indicator rather than a precise prescription tool. Future research should aim to refine these tools and test their utility in applied contexts with rigorous control over influencing factors.

Although the use of visual inspection may limit objectivity and reproducibility, it reflects the practical approach commonly used by coaches and practitioners when interpreting data from wearable devices. Our rationale was to test the feasibility of identifying the MFO zone with minimal data processing as would be the case in applied settings. Nevertheless, to address the concern regarding subjectivity, we also conducted segmented regression analysis as presented in Table 1. Although the agreement with the reference method remained limited, this comparison highlights the potential of integrating more objective computational methods in future applications.

Identify MFO is noteworthy due to its implications in the context of weight management (Dandanell, Boslev Praest et al. 2017; Dandanell, Husted et al. 2017), metabolic health and athletic performance (Wang et al. 2015; Chávez‐Guevara et al. 2020). As such, it may serve as a valuable marker for measurement. Traditionally, MFO has been detected via indirect calorimetry (Purdom et al. 2018; Amaro‐Gahete et al. 2019), a method that is both costly and complex. Furthermore, it is subject to limitations, such as protocol dependence (Amaro‐Gahete et al. 2019) and high daily variability (Chrzanowski‐Smith et al. 2020), rendering it inapplicable for routine use by athletes’ population. As it is well‐established, MFO is influenced by several factors including diet and physical exercise (Maunder et al. 2018). Consequently, changes in diet or regular endurance exercise can be expected to result in improvements in MFO (Maunder et al. 2018) leading to frequent misinterpretation of the data. In this regard, it may be of great importance to periodically evaluate MFO in order to adjust training intensity and ensure that a sufficient training stimulus and adaptations are maintained. The findings of this study indicate that it is feasible to detect the intensity range at which MFO happens utilising NIRS, reducing the cost of measurement compared to the traditional method and making periodic assessment feasible to carry out. Given the relatively short duration of the protocol (approximately 10 min for warm‐up and 16–24 min for testing), it may be feasible to evaluate MFO more frequently during training phases where the objective is to improve MFO. This would enable more frequent adjustments to training intensity (e.g., on a weekly basis) and nutritional interventions.

## 5 Limitations

The use of NIRS to assess muscle oxygenation is subject to inherent limitations that must be considered when addressing practical questions. This is evident in the present study and suggests that further research is necessary. The selection of muscle site and muscle heterogeneity are concerns when assessing a global response using a single NIRS probe (Koga et al. 2007; Okushima et al. 2015). The discordance between global exercise thresholds and local thresholds has been well‐documented in recent years and must be accounted for (Okushima et al. 2015; Caen and Boone 2023). Although lactate measurements were not included in this study, as the primary objective was to detect the MFO, indirect calorimetry serves as a more direct means of assessment (Bellotti et al. 2013; Batterson et al. 2023). Additionally, although acceptable filtering and modelling principles have been proposed for assessing SmO_2_ breakpoints, which should relate to measures, such as MFO, these principles have not been standardised. The appropriate NIRS parameters and calibration methods remain to be determined. The measurement protocol employed in this study was conducted in a laboratory setting, which may limit its applicability for home use. It would be of interest to investigate the potential for using a field protocol to detect the MFO zone utilising NIRS technology. Furthermore, NIRS is often compared to alternative metrics that themselves carry inherent limitations, resulting in a comparison between different approximating systems. Another important consideration is the duration of the fasting period prior to testing. Although participants refrained from caloric or stimulant intake for four hours before the assessment, it is possible that a longer fasting window (e.g., six hours) may have provided greater control over baseline metabolic conditions such as blood glucose and triglyceride levels. However, the selected duration was chosen to reflect common practice in exercise testing protocols and to balance metabolic control with participant comfort and adherence.

Moreover, the use of visual inspection to identify the SmO_2_ breakpoint, although reflecting real‐world application, introduces a degree of subjectivity that may limit reproducibility compared to computational approaches. Future studies may consider extending the fasting duration to minimise potential confounding effects on substrate oxidation. Finally, although our protocol aimed to reflect a realistic testing environment within a single laboratory visit, the absence of repeated testing sessions limits the assessment of intraindividual variability. Given the known day‐to‐day fluctuations in fat oxidation rates, future studies should incorporate multiday testing designs to better account for variability and enhance the robustness of NIRS‐based MFO zone detection.

We would like to highlight that, despite all the limitations we have mentioned and others that may be considered, the work we present is innovative and proposes a new direction for future research.

## 6 Conclusion

In conclusion, it is not possible to detect the MFO point using the NIRS device with the protocols and methods employed in this study. Disagreements were observed between the methods. However, it is possible to detect a general zone in which the MFO occurs. As such, coaches may use our proposed method to assess the MFO zone for the first time. Based on this general idea, a specific test should be conducted to assess the MFO. Further research is recommended to identify appropriate protocols that can enable the detection of the MFO point and to extrapolate to the field.

## Conflicts of Interest

Dr. Feldmann is a minority shareholder of, and professional contributor to Fortiori Designs LLC, the company that manufactures and distributes the NIRS device used in this study.

## Data Availability

The datasets generated and analysed during the current study are available from the corresponding author on reasonable request.
